# Characterization of quinazolinone calcilytic therapy for autosomal dominant hypocalcemia type 1 (ADH1)

**DOI:** 10.1016/j.jbc.2025.108404

**Published:** 2025-03-12

**Authors:** Fadil M. Hannan, Kreepa G. Kooblall, Mark Stevenson, Taha Elajnaf, Fangyu Liu, Kate E. Lines, Xin Meng, Michelle Stewart, Sara Wells, Edward F. Nemeth, Brian K. Shoichet, Michaela Kneissel, Juerg A. Gasser, Rajesh V. Thakker

**Affiliations:** 1 Nuffield Department of Women's & Reproductive Health, University of Oxford, Oxford, UK; 2 Academic Endocrine Unit, Radcliffe Department of Medicine, Oxford Centre for Diabetes, Endocrinology and Metabolism (OCDEM), University of Oxford, Oxford, UK; 3 Department of Pharmaceutical Chemistry, University of California, San Francisco, San Francisco, California, USA; 4 Mary Lyon Centre, MRC Harwell, Oxfordshire, UK; 5 MetisMedica, Toronto, Canada; 6 Disease Area for Diseases of Aging and Regenerative Medicine, Novartis Biomedical Research, Basel, Switzerland; 7 Centre for Endocrinology, William Harvey Research Institute, Barts and the London School of Medicine, Queen Mary University of London, London, UK

**Keywords:** genetic disease, parathyroid hormone, calcium-sensing receptor, molecular pharmacology, allosteric modulator, cell signaling, genetic mouse model

## Abstract

Gain-of-function mutations of the calcium-sensing receptor (CaSR) result in autosomal dominant hypocalcemia type 1 (ADH1), which may cause symptomatic hypocalcemia with low parathyroid hormone concentrations. Negative allosteric CaSR modulators, known as calcilytics, have potential as a targeted ADH1 therapy and comprise two main classes, which are the amino alcohols and the quinazolinones. Amino alcohol calcilytics have been assessed as ADH1 therapies but may not be effective for all ADH1-causing mutations. We therefore evaluated quinazolinone calcilytics (ATF936 and AXT914) as an alternate ADH1 treatment. Calcilytic docking studies were performed using reported cryo-EM CaSR structures. *In vitro* dose–response studies were performed using CaSR-expressing HEK293 cells and *in vivo* studies undertaken in mice with a gain-of-function CaSR mutation, Leu723Gln, known as *Nuf*. ATF936 and AXT914, as well as the amino alcohol calcilytics, NPS 2143 and NPSP795, were shown to bind at a common region within the CaSR transmembrane domain, which is also an ADH1 mutational hotspot. Treatment of cells expressing the *Nuf* mutant (Gln723) CaSR with 1 to 20 nM AXT914 caused dose-dependent decreases in CaSR-mediated intracellular calcium responses with 10 nM AXT914 normalizing the gain of function. Oral administration of 10 mg/kg AXT914 to *Nuf* mice increased parathyroid hormone to 104 ± 29 pmol/l compared with 23 ± 4 pmol/l for vehicle-treated mice, *p* < 0.05; and increased plasma albumin–adjusted calcium to 2.03 ± 0.02 mmol/l compared with 1.84 ± 0.02 mmol/l for vehicle-treated mice, *p* < 0.001. These studies indicate that quinazolinone calcilytics may have potential for treating ADH1.

Autosomal dominant hypocalcemia (ADH) is a monogenic disorder of systemic calcium homeostasis consisting of two types: ADH1 (OMIM #601198), which is caused by germline gain-of-function mutations of the extracellular calcium-sensing receptor (CaSR) (1, 2); and ADH2 (OMIM #615361), which is due to germline gain-of-function mutations of G-protein subunit alpha-11 (Gα_11_) (3, 4, 5). The CaSR is a highly expressed G-protein–coupled receptor (GPCR) in the parathyroid glands and kidneys (6). The CaSR and Gα_11_ proteins respond to elevations in extracellular calcium (Ca^2+^_e_) by increasing signaling *via* Gα_11_ that activates pathways, such as phospholipase C-mediated intracellular calcium (Ca^2+^_i_) oscillations, which decrease parathyroid hormone (PTH) secretion and promote renal calcium excretion, thereby normalizing extracellular calcium concentrations (6). ADH1 is the major disease type with a reported prevalence of 1:25,000 (7). This disorder is characterized by lifelong hypocalcemia, hypomagnesemia, hyperphosphatemia, low or inappropriately normal PTH concentrations, and inappropriately normal or increased urinary calcium excretion (1, 2, 8). These mineral disturbances may lead to neuromuscular and neurological symptoms, such as paresthesia, muscle spasms, and seizures in ∼50% of patients; and soft tissue calcifications affecting the basal ganglia in >35% of patients and kidneys in >10% of patients (1, 2, 8, 9). In addition, severe types of ADH1, which are typically associated with constitutively activating germline CaSR mutations, can cause a Bartter-like syndrome characterized by renal salt wasting leading to hypokalemic alkalosis and hyper-reninemic hyperaldosteronism (10, 11, 12).

ADH1 has a large unmet clinical need as commonly used treatments such as active vitamin D metabolites may cause excessive activation of the renal CaSR, thereby leading to severe hypercalciuria, renal calcifications, calculi, and impaired kidney function (1, 8). Recombinant PTH has also been used to manage ADH1 patients with hypocalcemic symptoms and is effective at ameliorating recurrent seizures in affected children and young adults (10). However, PTH is expensive, and its use is limited as this peptide has to be given by multiple daily injections or continuous infusion (10). More suitable therapies are required, and CaSR negative allosteric modulators (NAMs), which are also known as calcilytics, have potential as an ADH1 targeted therapy (13, 14). Calcilytics consist of two main classes of orally administered compounds: the amino alcohols and the quinazolinones (13, 14). The amino alcohol calcilytics have been extensively investigated as ADH therapies. Thus, the NPS 2143 (Fig. 1*A*), ronacaleret, NPSP795 (Fig. 1*B*), and encaleret amino alcohol compounds have been shown to rectify increased signaling responses associated with ADH1-causing mutant CaSRs *in vitro* (15, 16, 17, 18). Furthermore, NPS 2143, ronacaleret, NPSP795, and encaleret increased plasma calcium and PTH concentrations in ADH1 mouse models *in vivo* (15, 16, 19, 20). Moreover, phase 2 clinical studies have demonstrated that intravenous administration of NPSP795 increased PTH concentrations in five ADH1 patients, and oral administration of encaleret increased plasma calcium and PTH without causing hypercalciuria in 13 ADH1 patients (18, 21).

**Figure 1:**
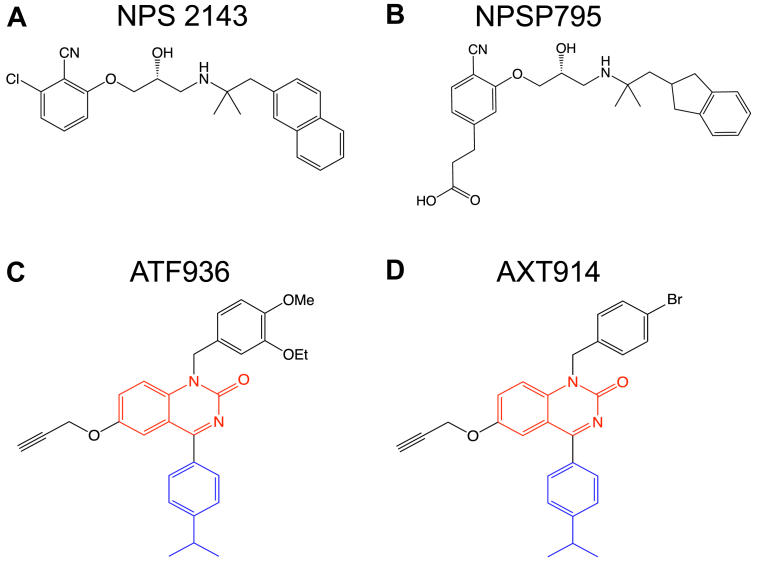
**Amino alcohol and quinazolinone calcilytic structures.** (*A*) NPS 2143; (*B*) NPSP795; (*C*) ATF936; and (*D*) AXT914. The quinazolinone and cumene (isopropylbenzene) rings of ATF936 and AXT914 are shown in *red* and *blue*, respectively. Structures are adapted with permission from Hannan *et al.* (13).

However, not all ADH1-causing mutations are responsive to amino alcohol calcilytics. For example, NPS 2143 only partially rectified the increased signaling responses caused by CaSR mutations associated with a Bartter-like syndrome (17). In addition, mutations of the CaSR Glu837 residue, which binds amino alcohol calcilytics, abrogated the activity of NPS 2143 and ronacaleret (22, 23). Thus, additional classes of calcilytics may be required to manage ADH1 patients harboring a variety of CaSR mutations, and we have therefore characterized the potential of quinazolinone calcilytics as an alternate ADH1 treatment. We utilized reported cryo-EM CaSR structures to delineate the predicted binding sites of two structurally similar quinazolinones, ATF936 and AXT914 (Fig. 1, *C* and *D*), and ascertain their proximity to residues mutated in ADH1 patients (24). AXT914 was further investigated as an ADH1 therapy, as this quinazolinone calcilytic has been previously demonstrated to be effective for treating rats with postsurgical hypoparathyroidism, which were generated by hemiparathyroidectomy or by total parathyroidectomy with autotransplantation of parathyroid tissue, and also to lead to sustained elevations in serum calcium concentrations in healthy human volunteers (25, 26). To evaluate the efficacy of AXT914 for ADH1, we undertook *in vitro* and *in vivo* studies involving the nuclear flecks (*Nuf*) mouse model, which has hypocalcemia in association with a germline gain-of-function CaSR mutation, Leu723Gln (27). We specifically selected the Leu723Gln mutation, as it causes a nonconstitutive mild-to-moderate CaSR gain of function that is representative of the functional effects of the majority of CaSR mutations causing ADH1 in humans (10, 18), and is also reported to be responsive, *in vitro* and *in vivo*, to several different amino alcohol calcilytics (NPS 2143, NPSP795, and ronacaleret) (15, 19, 20). Moreover, another missense mutation of the Leu723 residue (Leu723Arg) that was also associated with a CaSR gain of function has recently been reported in a patient with ADH1, thereby further underscoring the importance of the Leu723 residue in CaSR function and etiology of ADH1 (28).

## Results

### Quinazolinone and amino alcohol calcilytics likely share a common binding site within the CaSR transmembrane domain

**Figure 2:**
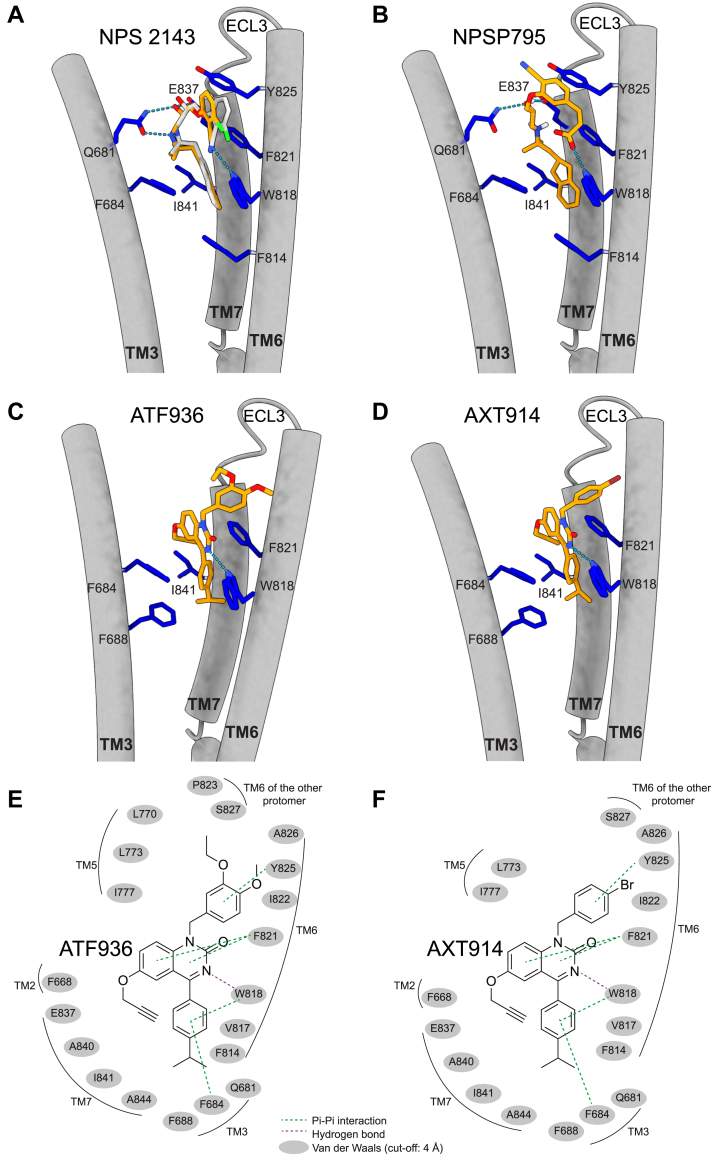
**Docking of amino alcohol and quinazolinone calcilytics within the CaSR transmembrane (TM) domain (TMD).***A*–*D,* diagram of the CaSR TMD calcilytic binding site, which is derived from a published cryo-EM structure (Protein Data Bank code: 7M3E) (24). *A,* binding site for NPS 2143 with the docked and reported cryo-EM poses shown in *light gray* and *orange*, respectively (24). *B*–*D,* docked poses of (*B*) NPSP795; (*C*) ATF936; and (*D*) AXT914, which are predicted to bind in a cavity formed by the extracellular portion of TM helices 3 (TM3), 6 (TM6), and 7 (TM7) (23). The poses shown for ATF936 and AXT914 are those with their most negative GlideScores of −8.6 and 8.5 kcal/mol, respectively. Residues involved in calcilytic binding are shown in *blue*. Heteroatoms are shown as follows: chlorine (*green*); oxygen (*red*); and nitrogen (*blue*). *Red regions* on amino acid side chains represent oxygen, and *light blue* regions represent nitrogen. Hydrogen bond ligand–receptor interactions are represented by *dashed lines*. ECL3, extracellular loop 3. *E* and *F,* 2-D structure of the calcilytic binding site showing residue interactions between the CaSR and (*E*) ATF936 and (*F*) AXT914 quinazolinone calcilytics. Hydrogen bonds are shown in *purple*, Pi–Pi interactions in *green*, and residues located within 4 Å and likely involved in van der Waals forces, a distance-dependent interaction between molecules, are shown in *gray*. CaSR, calcium-sensing receptor.

Five residues, which had previously been shown to impair CaSR binding affinity of the ATF936 quinazolinone calcilytic when mutated, were modeled using the cryo-EM structure of the inactive CaSR bound to NPS 2143 (Fig. 2) (23, 24). These five predicted quinazolinone calcilytic binding residues are all hydrophobic and located in transmembrane helix 3 (TM3) (Phe684, Phe688), TM6 (Trp818, Phe821), and TM7 (Ile841) (Fig. 2). This region within the CaSR transmembrane domain (TMD) overlaps with the amino alcohol calcilytic binding site as the Phe684, Trp818, Phe821, and Ile841 residues were also shown to bind NPS 2143 in reported cryo-EM CaSR structures (Fig. 2*A*) (24, 29). To model the interactions of ATF936 and AXT914 with the predicted binding residues, these calcilytics were docked to the NPS 2143 binding site using the Grid-based Ligand Docking with Energetics (Glide), Standard Precision mode, method that indicates stronger and more stable ligand–receptor interactions by more negative GlideScores (30). Initial docking evaluations were assessed with amino alcohol calcilytics, NPS 2143 and NPSP795 (Figs 1, *A* and *B* and 2, *A* and *B*), and this yielded favorable GlideScores of −9.9 and −10.1 kcal/mol, respectively. Docking analysis of NPS 2143 also showed a low RMSD of 0.94 Å (with RMSD ≤2.0 Å indicating acceptable similarity between two superimposed atomic coordinates) when compared with its reported cryo-EM pose (24). Docking evaluations of the quinazolinone calcilytics revealed that the 10 predicted poses of ATF936 and AXT914 with the most negative GlideScores had similar spatial locations and orientations within the calcilytic binding site, and with their cumene (isopropylbenzene) ring facing into the TMD (Fig. S1). The most favorable docking scores for the predicted poses of ATF936 and AXT914 were −8.6 and −8.5 kcal/mol, respectively (Fig. 2).

In order to exclude calcilytic poses with unfavorable binding free energies, we further assessed these docking poses with Glide Extra Precision mode (31). This yielded negative GlideScores of −9.0 and −10.2 kcal/mol for ATF936 and AXT914, respectively (Fig. S2). In addition, an assessment of the buried surface area of the calcilytics within the CaSR TMD showed that both ATF936 and AXT914 are well buried in the binding pocket (80% and 84%), similar to NPS 2143 (86%), suggesting that these ligand poses are credible (32, 33).

A structural analysis of ligand–receptor interactions showed that in their docked poses, ATF936 and AXT914 are predicted to have pi–pi, hydrogen bond, and van der Waal (vdW) interactions with residues within the NPS 2143 binding site (Fig. 2, *E* and *F*). Thus, the cumene ring of ATF936 and AXT914 forms pi–pi interactions with Phe684 and Trp818, which are a type of noncovalent interaction between unsaturated (poly) cyclic molecules, whereas the central quinazolinone ring forms pi–pi interactions with Phe821, and the phenol ring that with either bromide (AXT914) or O-methyl and O-ethyl (ATF936) groups forms a pi–pi interaction with Tyr825 (Fig. 2, *E* and *F*). In addition, the quinazolinone ring of ATF936 and AXT914 forms a hydrogen bond interaction with the binding site Trp818 residue (Figs. 2, *C*–*F* and S1). Furthermore, ATF936 and AXT914 are predicted to form vdW interactions with residues in TM2, TM3, TM5, TM6, and TM7 (Fig. 2, *E* and *F*). Thus, these findings have identified interactions involved in the binding of quinazolinone calcilytics to the CaSR TMD and highlight that the different calcilytic chemotypes bind at a similar site. However, there are likely differences in the binding of the quinazolinone and amino alcohol calcilytic chemotypes. For example, NPS 2143 and NPSP795 form bidentate hydrogen bonds with the polar Gln681 and negatively charged Glu837 residues (Fig. 2, *A* and *B*) (24, 29), whereas ATF936 and AXT914 may form vdW interactions with these residues (Fig. 2, *E* and *F*). Of note, engineered mutagenesis of Glu837 has demonstrated the importance of this residue for binding amino alcohol calcilytics, whereas substitution of Glu837 with an alanine residue was reported to have no significant effect on binding of ATF936 (Fig. 2, *E* and *F*) (23).

### ADH1 mutations cluster at the calcilytic binding site and affect residues involved in binding quinazolinone and amino alcohol calcilytics

**Figure 3:**
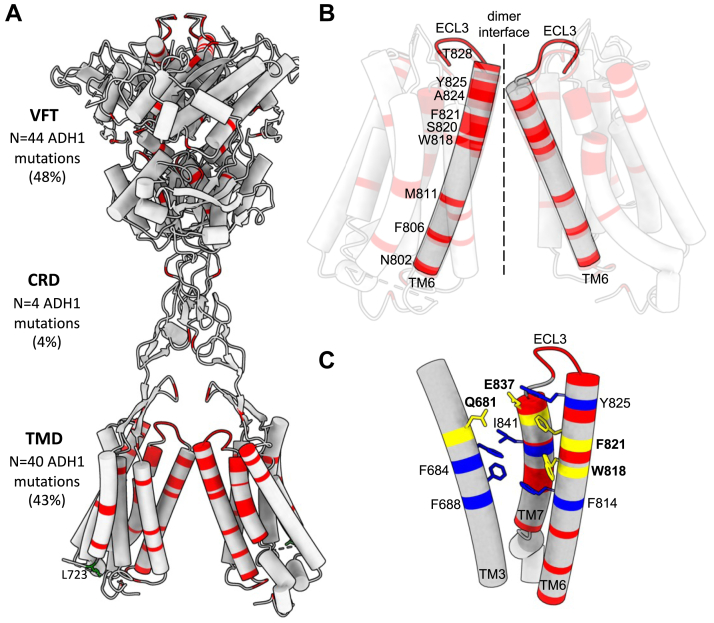
**Location of reported ADH1-causing CaSR mutations and proximity to the calcilytic binding site.** Active and inactive CaSRs were derived from published cryo-EM structures (PDB code: 7M3F and 7M3J, respectively). *A,* distribution of 93 reported ADH1 mutations (*red*) in the near full-length ligand-bound active CaSR dimer (PDB code: 7M3F) (24). The Leu723 (L723) residue, whose missense mutations Leu723Arg and Leu723Gln are associated with ADH1 in a patient (28) and the mouse model *Nuf* for ADH1, respectively, is shown in *green*. Five ADH1 mutations located in the CaSR intracellular domain could not be modeled because of lack of cryo-EM structures for this CaSR region. *B,* close-up view of the TMD region of the active CaSR showing location of residues at the TM6–TM6 dimer interface, which are mutated in ADH1 patients (*red*). *C,* close-up view of the calcilytic binding site in the inactive CaSR TMD (PDB code: 7M3J), showing that this region overlaps with the TM6–ECL3–TM7 ADH1 mutational hotspot. Residues mutated in ADH1 patients are shown in *red*. Calcilytic binding residues are shown in *blue* and labeled; and calcilytic binding residues that are also mutated in ADH1 patients are shown in *yellow* and labeled in *bold*. ADH1, autosomal dominant hypocalcemia type 1; CaSR, calcium-sensing receptor; CRD, cysteine-rich domain; ECL3, extracellular loop 3; PDB, Protein Data Bank; TMD, transmembrane domain; VFT, venus flytrap domain.

Missense substitutions account for >95% of all ADH1 mutations, and a total of 93 ADH1-causing missense mutations affecting 72 different CaSR residues were identified in the Human Gene Mutation Database (34, 35). An assessment of the distribution of these ADH1 missense mutations using the cryo-EM near full-length CaSR structure showed that 48 mutations (52%) were located in the extracellular venus flytrap and cysteine-rich domains, whereas 40 mutations (43%) were located in the TMD and five mutations (5%) were in the intracellular domain (Fig. 3*A*). More than 50% of the ADH1-causing TMD mutations clustered in a 45 amino acid contiguous region involving TM6, extracellular loop 3, and TM7 (Fig. 3*B*). This ADH1 mutational hotspot is located in a key region of the CaSR, which is involved in ligand-induced receptor dimerization (Fig. 3*B*) (24, 29). Moreover, the TM6–extracellular loop 3–TM7 region contributes to the calcilytic binding site, and ADH1 mutations have been reported to affect the TM6 Trp818 and Phe821 residues, which bind both ATF936 and NPS 2143, and also the TM7 Glu837 residue, which binds NPS 2143 (Fig. 3*C*).

### The AXT914 quinazolinone calcilytic decreases intracellular Ca^2+^ responses in cells expressing the gain-of-function mutant Gln723 CaSR

**Figure 4:**
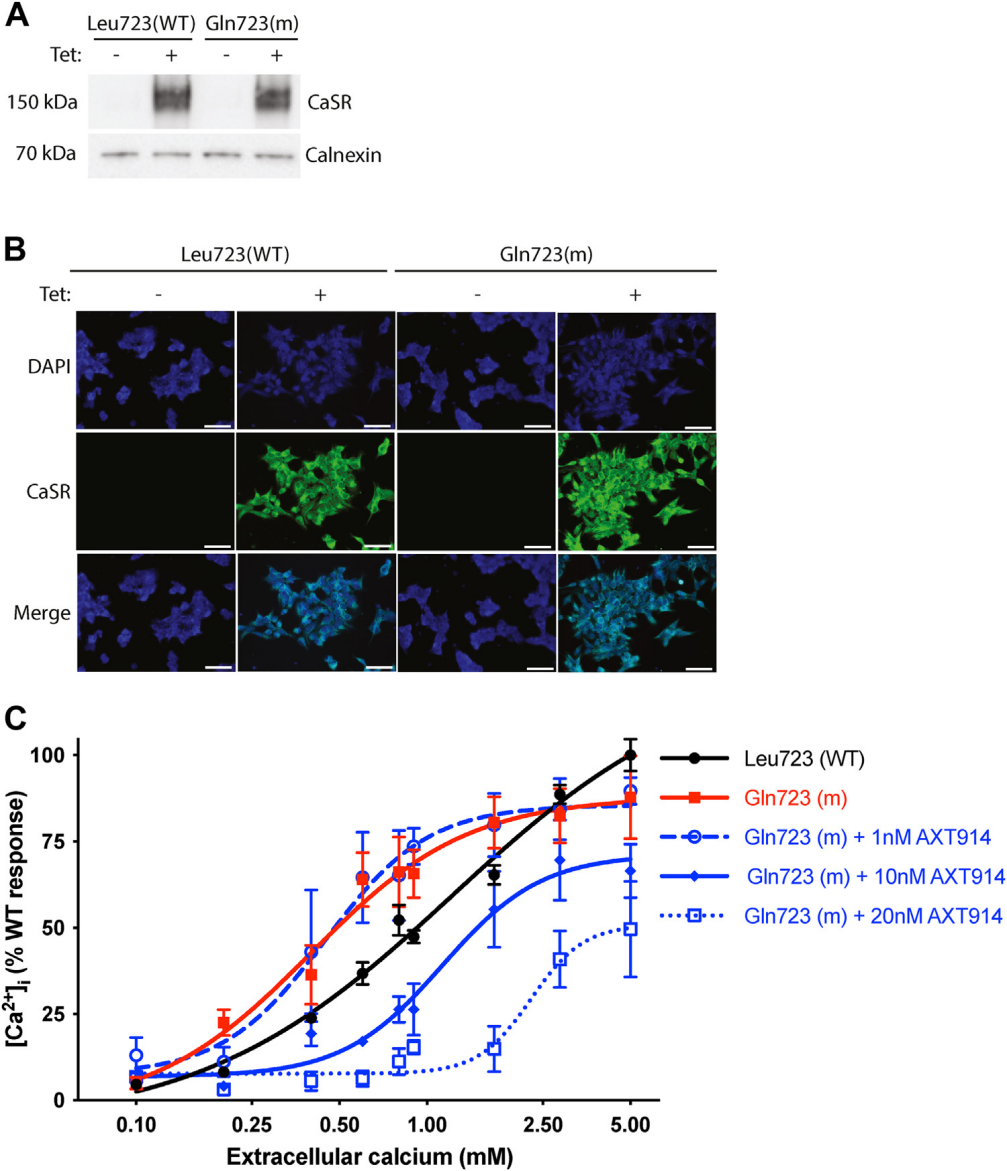
**Effect of AXT914 on the Ca^2+^_i_ responses of HEK293 cells stably expressing WT (Leu723) or mutant (Gln723) CaSR.***A,* Western blot analysis of lysates from T-REx-CaSR-WT and mutant T-REx-CaSR-Gln723 cells showing that tetracycline (Tet) induces CaSR protein expression. The *upper* and *lower* bands represent the mature glycosylated (∼160 kDa) and core glycosylated (∼140 kDa) forms of the CaSR, respectively (39). The calnexin housekeeping protein was used as a loading control. *B,* immunofluorescence analysis of T-REx-CaSR-WT and mutant T-REx-CaSR-Gln723 cells showing that Tet induces CaSR expression at the plasma membrane and within the cytoplasm. DAPI was used to stain cell nuclei. Scale bar represents 50 μm. *C,* Ca^2+^_i_ responses of T-REx-CaSR-WT and mutant T-REx-CaSR-Gln723 cells. The Ca^2+^_i_ responses to changes in Ca^2+^_e_ concentrations are expressed as a percentage of the maximum WT response. The Gln723 mutant CaSR caused a *leftward* shift in the concentration–response curve (*solid red line*) compared with WT (*solid black line*). The addition of 10 nM AXT914 rectified the *leftward* shift of the Gln723 mutant (*Nuf*) CaSR (*solid blue line*), whereas 20 nM AXT914 caused a marked *rightward* shift of the Gln723 mutant CaSR (*dotted line*), and 1 nM AXT914 did not alter the *leftward* shift of the Gln723 mutant CaSR (*dashed blue line*). Ca^2+^_i_, intracellular calcium; CaSR, calcium-sensing receptor; DAPI, 4′,6-diamidino-2-phenylindole; HEK293, human embryonic kidney 293 cell line.

We used HEK293 cells stably expressing the tetracycline-inducible CaSR (T-REx-CaSR) to assess whether the AXT914 quinazolinone calcilytic (Fig. 1*D*) can rectify the gain of function associated with the *Nuf* mouse Leu723Gln mutation, which is located at the cytoplasmic aspect of CaSR TM4 (Fig. 3*A*). Western blot analysis demonstrated that addition of tetracycline to the culture media led to the expression of the CaSR protein in cells harboring the WT (Leu723) and mutant Gln723 CaSR constructs (Fig. 4*A*). Tetracycline-induced CaSR protein expression was also confirmed by fluorescent microscopy (Fig. 4*B*). This demonstrated that WT and mutant CaSRs were expressed mainly at the plasma membrane and in the cytoplasm (Fig. 4*B*).

We next investigated the effect of AXT914 on Ca^2+^_e_-mediated Ca^2+^_i_ responses in the mutant Gln723 cells. Cells expressing the mutant Gln723 CaSR showed a leftward shift in the Ca^2+^_e_-mediated Ca^2+^_i_ concentration–response curve and a significantly reduced EC_50_ value of 0.40 mM (95% CI = 0.21–0.76 mM) when compared with the WT (Leu723) CaSR EC_50_ value of 1.22 mM (95% CI = 0.75–1.99 mM, *p* < 0.001) (Fig. 4*C* and Table 1), consistent with a CaSR gain of function, as reported (19, 20, 27). A dose titration of AXT914 in mutant Gln723 CaSR–expressing cells showed that 1 nM of this calcilytic had no effect on Ca^2+^_e_-mediated Ca^2+^_i_ responses (EC_50_ = 0.45 mM [95% CI = 0.29–0.70 mM]) (Fig. 4*C* and Table 1). In contrast, 10 nM of AXT914 caused a rightward shift of the mutant Gln723 receptor concentration–response curve and increased the EC_50_ value to 1.13 mM (95% CI = 0.79–1.61 mM), which was not significantly different from WT cells (Fig. 4*C* and Table 1), whereas 20 nM of AXT914 led to a marked rightward shift of the mutant Gln723 receptor curve and significantly increased the EC_50_ value compared with WT cells (Fig. 4*C* and Table 1). Thus, 10 nM of AXT914 was shown to normalize the gain of function caused by the *Nuf* mutant Gln723 CaSR. A concentration titration of AXT914 also decreased the maximal effect (E_max_) of Ca^2+^_e_ on the Ca^2+^_i_ response by around 50% whilst progressively increasing the Hill slope of the Ca^2+^_e_-mediated Ca^2+^_i_ concentration–response curves in mutant Gln723 CaSR–expressing cells (Fig. 4*C* and Table 1).

**Table 1:** Effect of AXT914 on the EC_50_, maximal effect (E_max_), and Hill slope of HEK293 cells stably expressing WT (Leu723) or mutant (Gln723) CaSR

Construct and dose	EC_50_ (95% CI)	E_max_ (% WT response)	Hill slope
Leu723 (WT)	1.22 (0.75–1.99)	100 ± 5	1.0 ± 0.3
Gln723 (m)	0.40 (0.21–0.76)∗∗	88 ± 12	1.6 ± 0.7
Gln723 (m) + 1 nM AXT914	0.45 (0.29–0.70)∗∗	90 ± 4	2.5 ± 1.2
Gln723 (m) + 10 nM AXT914	1.13 (0.79–1.61)	66 ± 8∗	2.6 ± 1.0
Gln723 (m) + 20 nM AXT914	2.27 (1.64–3.15)∗∗∗	50 ± 14∗∗∗∗	4.8 ± 2.6

Values are shown as mean with either SEM or 95% CI and were obtained from n = 4 experiments. ∗p < 0.05, ∗∗p < 0.01, ∗∗∗p < 0.001, ∗∗∗∗p < 0.0001 compared with cells expressing the WT (Leu723) CaSR.

### AXT914 increases plasma calcium and PTH in *Nuf* mice

**Figure 5:**
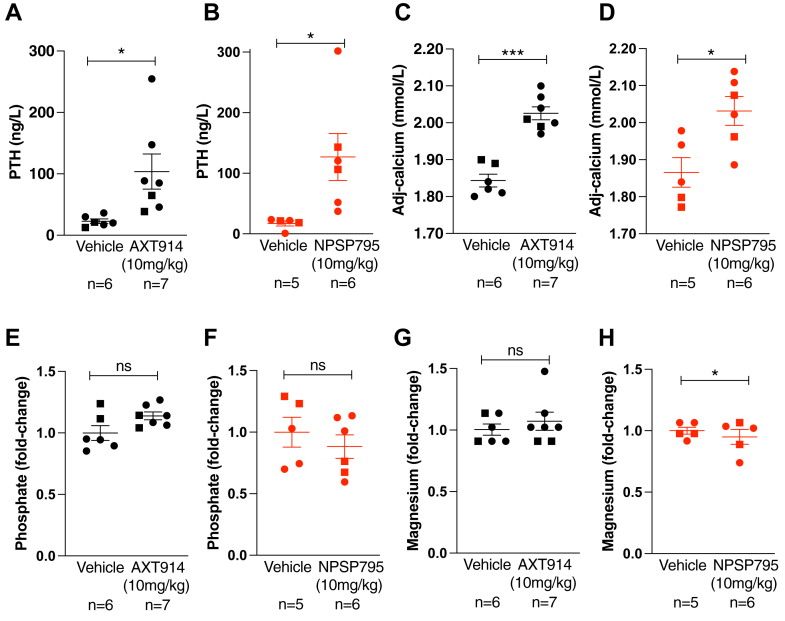
**Effect of AXT914 on plasma PTH, calcium, phosphate, and magnesium concentrations in *Casr*^*+/Nuf*^ mice and comparison with the NPSP795 amino alcohol calcilytic.** Plasma concentrations of (*A* and *B*) PTH, (*C* and *D*) albumin-adjusted calcium (adj-calcium), (*E* and *F*) phosphate, and (*G* and *H*) magnesium are shown. Increases in plasma PTH and adj-calcium concentrations were observed without alterations in plasma phosphate and magnesium concentrations in *Casr*^*+/Nuf*^ mice treated with either 10 mg/kg doses of orally administered AXT914 (*black*) or subcutaneously administered NPSP795 (*red*). Male and female mice are represented by *squares* and *circles*, respectively. Mean ± SEM values for the respective groups are indicated by *solid bars*. ∗*p* < 0.05; ∗∗∗*p* < 0.001; ns, not significant. Normal plasma concentration ranges (mean ± 2SD) for WT (*Casr*^*+/+*^*)* mice, derived from published data (19), are as follows: PTH, 0 to 153 ng/l; adj-calcium, 2.33 to 2.63 mmol/l; and phosphate, 0.72 to 1.84 mmol/l. Normal range for plasma magnesium concentration in WT mice is not available. PTH, parathyroid hormone.

As AXT914 rectified the altered Ca^2+^_e_-mediated Ca^2+^_i_ responses associated with the *Nuf* mouse CaSR mutation (Leu723Gln) *in vitro*, we proceeded to an *in vivo* assessment of this calcilytic in *Casr*^*+/Nuf*^ mice, which have hypocalcemia and reduced plasma PTH concentrations (Table 2). AXT914 was administered as a single 10 mg/kg oral bolus. This dose and route of administration was used as it has been previously shown to increase PTH both in WT rats (Fig. S3) and a rat postsurgical hypoparathyroidism model (26). To determine whether AXT914 may influence plasma mineral parameters similarly to that reported for amino alcohol calcilytics, the effects of this quinazolinone calcilytic were compared with *Casr*^*+/Nuf*^ mice previously treated with the equivalent (10 mg/kg) dose of the NPSP795 amino alcohol calcilytic administered as a subcutaneous bolus, as reported (19). Some of the data obtained with administration of 10 mg/kg of NPSP795 were not included for publication in the previous study (19). Administration of AXT914 to *Casr*^*+/Nuf*^ mice led to a significantly increased plasma PTH concentration of 104 ± 29 ng/l in treated mice at 30 min postdose compared with 23 ± 4 ng/l for control mice (*p* < 0.05) (Table 2 and Fig. 5*A*). This PTH response is similar to that reported for NPSP795-treated *Casr*^*+/Nuf*^ mice (PTH of 127 ± 39 ng/l in treated mice at 30 min postdose compared with 17 ± 4 ng/l for control mice) (19) (Fig. 5*B*). AXT914 also significantly increased plasma albumin–adjusted calcium at 2 h postdose in treated mice compared with controls (2.03 ± 0.02 mmol/l *versus* 1.84 ± 0.02 mmol/l, *p* < 0.0001) (Table 2 and Fig. 5*C*). This 10% increase in albumin-adjusted calcium was similarly observed in NPSP795-treated *Casr*^*+/Nuf*^ mice (Fig. 5*D*). Single-dose administration of AXT914 was well tolerated and did not significantly alter plasma concentrations of phosphate, magnesium, electrolytes, or renal function, similar to that in *Casr*^*+/Nuf*^ mice treated with NPSP795 (Table 2 and Fig. 5, *E*–*H*).

**Table 2:** Age and plasma biochemistry of *Casr*^*+/Nuf*^ mice given drug vehicle or AXT914

Parameter	*Casr*^*+/Nuf*^ mice	
	Vehicle-only group	AXT914 group
Number of mice	2 M, 4 F	2 M, 5 F
Age (months)	13.4 ± 0.2	13.3 ± 0.2
Total calcium (mmol/l)	1.74 ± 0.01	1.92 ± 0.01∗∗∗∗
Albumin-adjusted calcium (mmol/l)	1.84 ± 0.02	2.03 ± 0.02∗∗∗
Parathyroid hormone (ng/l)	23 + 4	104 + 29∗
Phosphate (mmol/l)	3.6 ± 0.2	4.1 ± 0.1
Magnesium (mmol/l)	0.88 ± 0.04	0.94 ± 0.07
Alkaline phosphatase activity (U/l)	79 ± 16	78 ± 10
Sodium (mmol/l)	149 ± 0.6	148 ± 0.4
Potassium (mmol/l)	4.6 ± 0.2	4.5 ± 0.1
Chloride (mmol/l)	109 ± 1	107 ± 1
Urea (mmol/l)	16.1 ± 1	14.4 ± 1
Creatinine (μmol/l)	14.3 ± 0.4	13.2 ± 0.8

F, female; M, male.

∗*p* < 0.05, ∗∗∗*p* < 0.001, ∗∗∗∗*p* < 0.0001 compared with control *Casr*^*+/Nuf*^ mice. Data are shown as mean ± SEM. Normal plasma concentration ranges (mean ± 2SD) for WT (*Casr*^*+/+*^*)* mice, derived from published data (19) are as follows: albumin-adjusted calcium, 2.33 to 2.63 mmol/l; parathyroid hormone, 0 to 153 ng/l; phosphate, 0.72 to 1.84 mmol/l; alkaline phosphatase activity, 40 to 156 U/l; urea, 4.7 to 11.9 mmol/l; and creatinine, 5.9 to 20.2 μmol/l. Normal ranges for plasma magnesium, sodium, potassium, and chloride concentrations in WT mice are not available.

## Discussion

The findings from this proof-of-efficacy study have shown that the quinazolinone calcilytic, AXT914, acts in a concentration-dependent manner, *in vitro*, to progressively increase the EC_50_ and decrease the E_max_ associated with the *Nuf* mouse CaSR mutation, Leu723Gln, thereby rectifying its gain of function (Fig. 4). AXT914 also increased the steepness of the Ca^2+^_e_-mediated Ca^2+^_i_ concentration–response curves, as indicated by the higher Hill slope values (Table 1). Calcimimetics that act as CaSR-positive allosteric modulators are reported to have the opposite effect of decreasing the Hill slope in CaSR-expressing cells (36). Such effects on the Hill slope have been postulated to be due to allosteric modulators either affecting cooperative binding of Ca^2+^ to the CaSR or influencing the effects of CaSR agonists that are present at basal concentrations in the cell culture media (36).

Administration of AXT914 also increased plasma PTH and calcium concentrations, *in vivo* in *Nuf* mice, which are a model for ADH1 (Fig. 5). Previous rat studies have demonstrated that AXT914 has around 30% bioavailability when orally administered (Table S1). Despite having limited bioavailability, a 10 mg/kg oral AXT914 dose increased plasma concentrations of PTH and albumin-adjusted calcium to a similar degree to that observed with the equivalent subcutaneously administered dose of a different calcilytic chemotype, NPSP795, thereby demonstrating the *in vivo* efficacy of this quinazolinone calcilytic (Fig. 5). It is likely that these effects of AXT914 are mediated specifically by the CaSR, as previous *in vitro* radiolabeled ligand-binding studies have shown little or no crossreactivity of AXT914 with a panel of cell surface proteins consisting of GPCRs other than the CaSR and also ion channels and transporters (Table S2). Moreover, the single-dose administration of AXT914 showed no significant effect on plasma phosphate and magnesium, which are frequently altered in ADH1 patients (10). Longer-term repetitive dosing studies are required to determine whether AXT914 may rectify these mineral disturbances and also ameliorate the hypercalciuria associated with ADH1. In support of this, once-daily dosing of 10 mg/kg AXT914 over a 2-week period was reported to decrease both serum phosphate and the urine calcium-to-creatinine ratio in rats with postsurgical hypoparathyroidism (26). Longer-term studies are also warranted to assess whether repetitive AXT914 dosing may induce sustained elevations in circulating calcium concentrations in the setting of ADH1. In keeping with this, healthy postmenopausal women dosed with 120 mg AXT914 once daily for 12 days were reported to have increases in serum calcium concentrations that lasted for up to 48 h after the final dose (25). In addition, postsurgical hypoparathyroid rats treated with AXT914 for 21 days continued to have elevations in serum calcium and PTH for 7 days after this calcilytic had been discontinued (26).

Quinazolinone calcilytics are potent CaSR NAMs, and consistent with this, 10 nM of AXT914 normalized Ca^2+^_i_ responses in cells expressing the mutant *Nuf* CaSR (Fig. 4*C*), whereas higher (≥20 nM) doses of the NPS 2143, NPSP795, and ronacaleret amino alcohol calcilytics were reported to be required to rectify gain of function caused by this mutant CaSR (15, 19, 20). An allosterism model that utilizes CaSR-mediated Ca^2+^_i_ responses has provided insights into the pharmacological basis of this potency (23, 37). This model predicted that quinazolinone calcilytics have a higher binding affinity for the CaSR compared with amino alcohol calcilytics (23). Moreover, quinazolinones have greater negative cooperativity, indicating that at equivalent degrees of receptor occupancy, these compounds cause greater inhibition of CaSR responsiveness to Ca^2+^_e_ compared with amino alcohol calcilytics (23). In keeping with this, *in vitro* studies have shown that AXT914 can rectify the severe or constitutive gain of function associated with CaSR mutant proteins causing ADH1 with a Bartter-like syndrome, whereas these mutations were only partially responsive to NPS 2143 treatment (17).

Mutagenesis studies and structural analyses involving CaSR TMD homology models based on the class A rhodopsin and class C metabotropic glutamate GPCRs have previously predicted that the quinazolinone and amino alcohol calcilytics, which are structurally distinct (Fig. 1), bind at a similar site within a cavity formed by the CaSR transmembrane helices (23, 38). We have modeled quinazolinone calcilytic binding using the cryo-EM structure of the inactive CaSR bound to the NPS 2143 amino alcohol calcilytic and shown a substantial overlap in the binding sites of these two calcilytic chemotypes (Fig. 2). However, there are key differences as NPS 2143 interacts with hydrophobic and polar or charged residues, whereas the ATF936 quinazolinone calcilytic is predicted to only bind hydrophobic residues (23, 24). These findings highlight that quinazolinone and amino alcohol calcilytics likely exert antagonist effects on CaSR function by interacting with distinct amino acid networks within the TMD (23).

We also characterized the location and distribution of reported ADH1 missense mutations using reported cryo-EM CaSR structures (24). Our analysis showed a clustering of mutations along the transmembrane dimer interface (Fig. 3), and it is possible that these ADH1-causing missense substitutions induce a gain of function by enhancing CaSR dimerization upon ligand binding. ADH1 mutations were additionally shown to be located at the calcilytic binding site and affect residues that selectively interact with NPS 2143 or interact with both NPS 2143 and ATF936 (Fig. 3), although the *Nuf* mouse–associated Leu723Gln mutation is located outside the calcilytic binding pocket (Fig. 3), and is therefore less likely to affect the actions of these NAMs. These findings highlight that the location and structure–function consequences of the underlying mutation will need to be considered when selecting ADH1 patients for calcilytic treatment. Moreover, different calcilytic chemotypes will likely be required to provide a comprehensive precision medicine-based approach for successful treatment of ADH1.

In summary, our *in silico*, *in vitro*, and *in vivo* studies highlight that the quinazolinone class of calcilytics have potential as a targeted therapy for ADH1 patients.

## Experimental procedures

### Compounds

AXT914 was provided by Novartis Pharma AG. An AXT914 formulation was prepared using a microemulsion preconcentrate consisting of Cremophor RH40 (36% w/w, Sigma–Aldrich), Capmul MCM C8 (54% w/w, Abitec), and triethylcitrate (9% w/w, Sigma–Aldrich), which was then made up to the required volume using 10% ethanol (Sigma–Aldrich) prior to administration in *in vitro* and *in vivo* studies. This microemulsion of AXT914 was formulated prior to gavage dosing as quinazolinone calcilytics are highly lipophilic and have limited bioavailability when administered orally (38). Use of microemulsions has been shown to optimize the pharmacokinetic properties of this class of calcilytics and increase their peak serum concentrations following oral dosing (38). NPSP795 was provided by NPS/Shire Pharmaceuticals and dissolved in a 20% aqueous solution of 2-hydroxypropyl-β-cyclodextrin (Sigma–Aldrich) for use in *in vivo* studies (19). All NPSP795 data were generated for a published report (19), with the plasma calcium, phosphate, and magnesium data described in the current study being previously unpublished.

### Animals

*Nuf* mice (symbol: *Casr*^*Nuf*^; Mouse Genome Informatics [MGI] ID: MGI:3054788) were kept as a closed colony on the inbred 102/H background (MGI:5291924), which is a substrain originally bred at the Medical Research Council (MRC) Harwell Centre (19). Mice were maintained according to UK Home Office guidance at the MRC Harwell Centre in an environment controlled for temperature (21 ± 2 °C), humidity (55 ± 10%), and light (12 h light–dark cycle) (19). Mice were fed *ad libitum* on a commercial diet (RM3; Special Diet Services) that had 1.24% calcium, 0.83% phosphorus, and 2948 IU/kg of vitamin D and were also given free access to water (25 ppm chlorine) (19). Study mice were aged between 12 and 14 months and obtained from eight litters generated from five matings.

### Stable cell lines

HEK293 cells stably expressing the tetracycline-inducible WT and mutant CaSR (T-REx-CaSR-WT and T-REx-CaSR-Gln723, respectively) were previously generated using the T-REx-HEK293 Flp-In cell-line system (Invitrogen), as reported (19). Cells were maintained in Dulbecco's modified Eagle's medium Glutamax media (Gibco) supplemented with 10% heat-inactivated fetal bovine serum (Gibco), 2 mM glutamine (Gibco), 10 μg/ml blasticidin (Invitrogen), and 100 μg/ml hygromycin B (Invitrogen) (19). All cells were incubated at 37 °C, 5% CO_2_, and 95% humidity. Twenty-four hours prior to performance of *in vitro* assays, expression of WT and mutant Gln723 CaSR proteins was induced using 1 μg/ml tetracycline (Invitrogen) (19).

### Western blot analysis

Total protein was extracted from CaSR-expressing cells using ice-cold lysis buffer (150 mM NaCl, 50 mM Tris [pH 8.0], 1% Triton X-100 [v/v], and 1x Protease inhibitor tablet [Roche]), maintained in constant agitation at 4 °C for 30 min, centrifuged for 10 min at 10,000 rpm, and the supernatant collected. Protein concentration was determined using Bradford assay, and protein samples were prepared in 4x Laemmli loading dye (Bio-Rad), and resolved using precast 4 to 8% SDS-PAGE gel electrophoresis (Bio-Rad). Samples were transferred onto polyvinylidene difluoride membrane (PerkinElmer), blocked in 5% milk in PBS–Tween (PBS-T) and incubated with 1:2000 mouse anti-CaSR (MAI-934; Invitrogen) or 1:5000 rabbit anti-Calnexin (AB-2301; Millipore) antibodies in 5% milk/PBS-T. Membranes were subsequently incubated with 1:2000 anti-mouse or anti-rabbit horseradish peroxidase–conjugated secondary antibodies (SC-2004; Santa Cruz Biotechnology), respectively, and visualized using ECL Western blotting substrate (Bio-Rad) on a Chemidoc XRS+ system (Bio-Rad).

### Immunocytochemistry

To visualize CaSR expression in cells, glass coverslips were placed in 12-well plates (Corning) and coated with poly-d-lysine (Sigma) before adding stably expressing T-REx-CaSR-WT and mutant T-REx-CaSR-Gln723 cells. Cells were fixed on coverslips using ice-cold 100% methanol for 10 min prior to blocking with 1% bovine serum albumin in PBS-T for 30 min at room temperature. The cells were incubated with 1:100 mouse anti-CaSR primary antibody (MAI-934; Invitrogen) followed by 1:500 anti-mouse Alexa Fluor 488 secondary antibody (Invitrogen). ProLong Gold Antifade reagent (Invitrogen) was used to mount the coverslips for microscopy studies (Leica microscope model DM4000B and DFC320 digital camera; Leica Microsystems).

### Ca^2+^_i_ analysis

The Ca^2+^_i_ responses of CaSR-expressing cells were measured using Fluo-4 (Invitrogen), a cell-permeable fluorescent Ca^2+^ indicator, as described (7, 39). Briefly, T-REx-CaSR-WT and mutant T-REx-CaSR-Gln723 cells were seeded into black 96-well plates (Corning) coated with poly-d-lysine (7). Cells were treated with serum-free media overnight. Fluo-4 dye was prepared according to the manufacturer's instructions (Invitrogen), and cells were loaded for 1 h at 37 °C (7). Baseline measurements were made, and increasing concentrations of CaCl_2_ (0.1–5 mM) or 50 μM ionomycin (positive control) were injected automatically into each well. Changes in Ca^2+^_i_ were recorded on a PHERAstar instrument (BMG Labtech) at 37°C with an excitation filter of 485 nm and an emission filter of 520 nm (7). The peak mean fluorescence ratio of the transient response following each individual stimulus was measured using MARS data analysis software (BMG Labtech). Relative fluorescence units were normalized to the fluorescence stimulated by ionomycin to account for differences in cell number and loading efficiency and then further normalized to the maximum observed WT CaSR response.

### Administration of AXT914 to *Nuf* mice

Aged-matched adult male and female heterozygous (*Casr*^*+/Nuf*^) mice were randomly allocated to receive vehicle or 10 mg/kg AXT914 as a single bolus by oral gavage (40). No experimental procedures were conducted on the mice prior to drug administration. The study team were blinded during calcilytic dosing, sample collection, and when performing end-point assays. The primary study outcome was an increase in plasma albumin–adjusted calcium at 2 h after drug administration in *Casr*^*+/Nuf*^ mice.

### Biochemical measurements

Blood samples were obtained from the lateral tail vein using EDTA capillary blood collection tubes (Sarstedt) following administration of topical local anesthesia for analysis of plasma PTH. Blood was also obtained from the retro-orbital vein under isoflurane terminal anesthesia using lithium heparin capillary blood collection tubes (Sarstedt) for other biochemical analyses (19, 40). Samples were centrifuged at 5000*g* for 10 min at 8 °C, and plasma was separated for analysis of total calcium, albumin, phosphate, magnesium, alkaline phosphatase activity, sodium, potassium, chloride, urea, and creatinine on a Beckman Coulter AU680 analyzer, as reported (19, 40). Plasma calcium was adjusted for variations in plasma albumin as follows: (plasma calcium (mmol/l) – [(plasma albumin (g/l) – 30) x 0.02]) (19, 40). Plasma PTH was assayed using an ELISA for mouse intact PTH (Immutopics) (19, 40).

### CaSR structural analysis

Residues involved in calcilytic binding were modeled using a reported cryo-EM structure of the near full-length inactive CaSR bound to NPS 2143 (Protein Data Bank [PDB] code: 7M3E) (24). Residues mutated in ADH1 patients were obtained from Human Gene Mutation Database and modeled using cryo-EM structures of the active and inactive CaSR (PDB code: 7M3F and 7M3J, respectively) (24, 35). Structural analysis was performed using the University of California, San Francisco ChimeraX molecular visualization program (https://www.cgl.ucsf.edu/chimerax/) (41).

### Calcilytic docking analysis

Molecular docking was performed using Glide (Schrödinger, LLC, 2024) (30). 3D structures of the CaSR protein and calcilytics were prepared for molecular docking, as follows. A high-resolution cryo-EM structure of CaSR bound to NPS 2143 (PDB code: 7M3E; resolution: 3.20 Å) was imported to Protein Preparation Wizard in Maestro (Schrödinger, LLC, 2024, version 13.1.141, Release 2022-1) for: (1) protonation of the CaSR structure to generate tautomers with a target pH of 7.0 ± 2.0 using Epik (Schrödinger, LLC, 2024); (2) optimization of the protein H-bond network; and (3) restrained minimization, whereby heavy atom coordinates were unmodified with default parameters maintained for all other settings (42). 3D inputs of the calcilytic ligands used for Glide molecular docking were generated using LigPrep (Schrödinger, LLC, 2024), starting from a Simplified Molecular Input Line Entry System (SMILES) representation of each molecule followed by generation of tautomers with a target pH of 7.0 ± 2.0 using Epik, with a maximum of four stereoisomers produced per ligand. Subsequently, based on the minimized protein structure, docking grids were generated by Receptor Grid Generation using default parameters, with the NPS 2143 binding pocket set as the centroid of the docking box. The docking calculations were performed using both “Standard Precision (SP)” and “Extra Precision (XP)” modes of Glide docking (30). Glide utilizes docking scoring functions that incorporate contributions from lipophilic, H-bond, van der Waals, and electrostatic interactions between ligand and binding pocket as well as incorporating desolvation penalties, as described (30, 31). Ligand binding free energy is approximated using the GlideScore (kcal/mol) with more negative scores indicating stronger and more stable receptor–ligand complexes. Glide conducts an approximate systematic search for the positions, orientations, and conformations of each ligand within the receptor's binding site by employing a series of hierarchical filters. In the SP mode, 5000 poses per ligand were generated during the initial phase of the docking calculation and the best 400 poses per ligand were chosen for energy minimization. The energy minimization protocol included dielectric constant of 2.0 and 100 minimization steps. In the XP mode, 5000 poses per ligand were generated during the initial phase of the docking calculation and the best 800 poses per ligand were chosen for energy minimization. Upon completion of each docking calculations, a maximum of 10 top poses with the most negative GlideScores were generated and presented for each ligand. The buried surface areas of NPS 2143, ATF936, and AXT914 were calculated using PDBePISA (pdbe.org/pisa) by using the cryo-EM structure of CaSR in complex with NPS 2143 (PDB code: 7M3E) or with docked ATF936 or AXT914 as inputs, as reported (43).

### Statistical analyses

The *in vitro* Ca^2+^_i_ data were analyzed by nonlinear regression of Ca^2+^_e_ concentration–Ca^2+^_i_ response curves performed with GraphPad Prism (GraphPad Software, Inc) and using the Ca^2+^_i_ response at each Ca^2+^_e_ concentration normalized to the maximum WT response, as reported (44), for the determination of the following parameters: half-maximal concentration (EC_50_) (*i.e.*, Ca^2+^_e_ concentration inducing 50% of the maximal Ca^2+^_i_ response); the maximal effect (E_max_) (*i.e.*, maximum Ca^2+^_i_ response expressed as a percentage of the maximum WT Ca^2+^_i_ response); and the Hill slope that indicates the steepness of the Ca^2+^_e_ concentration–Ca^2+^_i_ response curves. Statistical comparisons of the *in vitro* Ca^2+^_i_ data were undertaken using the *F*-test (19). All *in vitro* studies were performed in four biological replicates with each replicate representing an independent experiment performed on a different day and using a different preparation of cells. Each independent experiment had three technical replicates. G∗Power statistical software was used to calculate mouse sample sizes (19). An individual mouse represented the unit of analysis. A minimum sample size of n = 5 mice allocated to the treatment and vehicle-only groups gave >80% power to detect a >15% increase in albumin-adjusted plasma calcium, as described (19). Statistical comparisons of the *in vivo* data were performed using the unpaired Student's *t* test, and all biochemical parameters were analyzed using GraphPad Prism. A value of *p* < 0.05 was considered significant. *In vitro* data are shown as mean with 95% CI or mean ± SEM, and *in vivo* data are shown as mean ± SEM.

## Data availability

All data are contained with the article.

## Supporting information

This article contains supporting information.

## Ethics statement

All procedures involving mice were approved by the MRC Harwell Institute Ethical Review Committee and licensed under the Animal (Scientific Procedures) Act 1986, which was issued by the UK Home Office (PPL30/2752).

## Conflict of interest

The authors declare that they have no conflicts of interest with the contents of this article.

## Supporting information

Table S2

NPSP795-CaSR structure

ATF936-CaSR structure

AXT914-CaSR structure
