# Supplementary material for: Characterization of quinazolinone calcilytic therapy for autosomal dominant hypocalcemia type 1 (ADH1)
Source: J Biol Chem. 2025 Mar 12;301(4):108404. doi: 10.1016/j.jbc.2025.108404 (PMC12001111; doi:10.1016/j.jbc.2025.108404)
Supplement: Figure S1 [file mmc1.pdf]

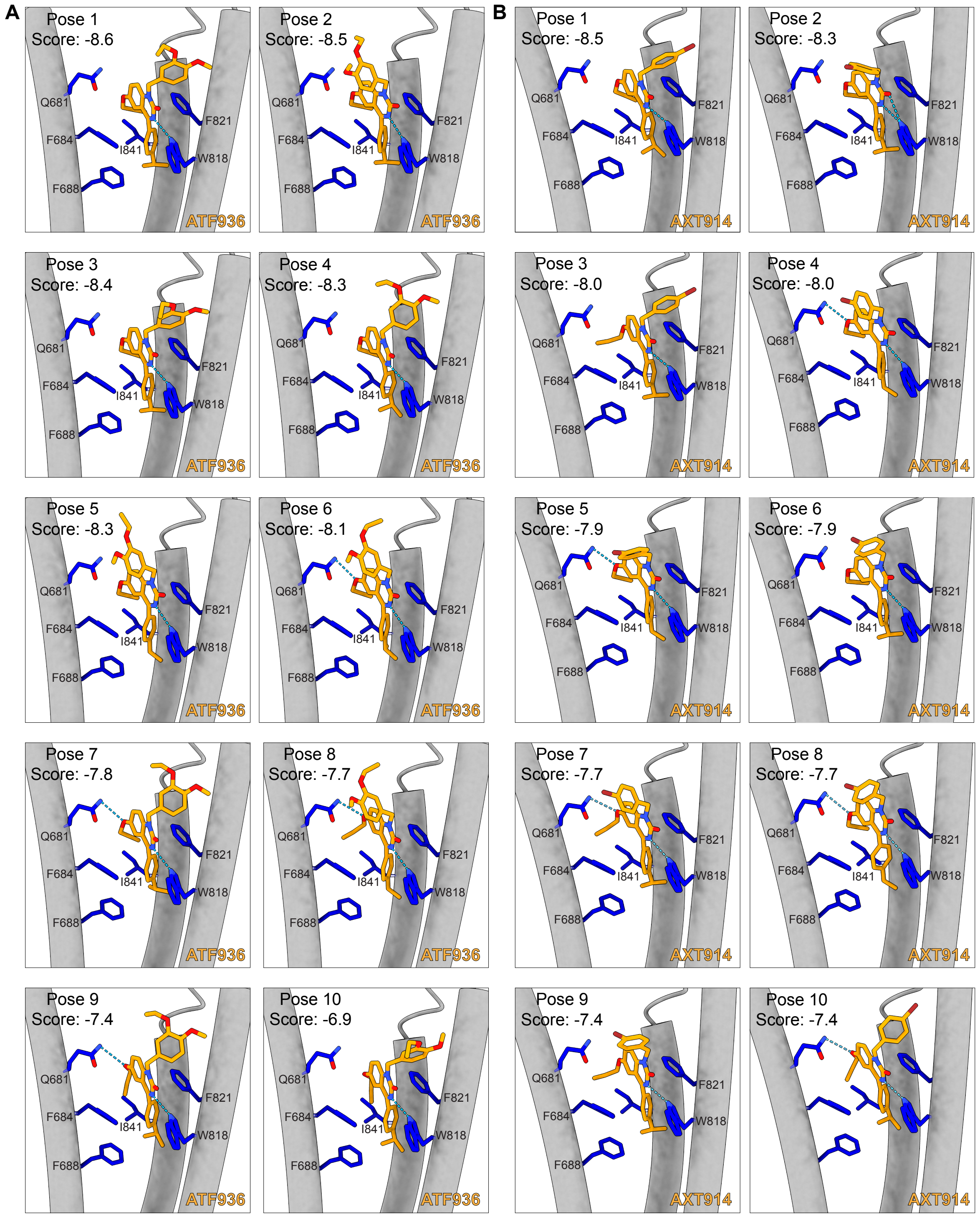

Figure S1. Top 10 docked poses of A) ATF936 and B) AXT914 quinazolinone calcilytics within the CaSR transmembrane domain. GlideScores are indicated for each pose. Residues involved in calcilytic binding are shown in blue. Calcilytic molecules are shown in orange with oxygen and nitrogen heteroatoms shown in red and blue, respectively. H-bond ligand-receptor interactions are represented by dashed lines.
