# Supplementary material for: Characterization of quinazolinone calcilytic therapy for autosomal dominant hypocalcemia type 1 (ADH1)
Source: J Biol Chem. 2025 Mar 12;301(4):108404. doi: 10.1016/j.jbc.2025.108404 (PMC12001111; doi:10.1016/j.jbc.2025.108404)
Supplement: Figure S2 [file mmc2.pdf]

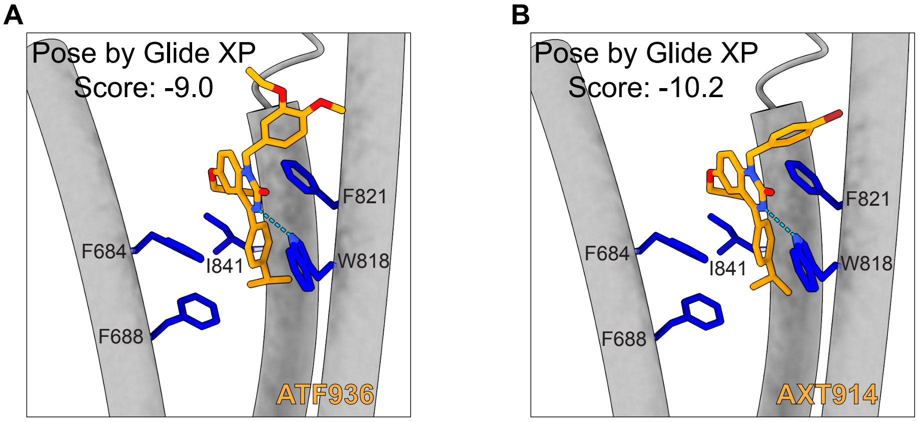

Figure S2. Docked poses of A) ATF936 and B) AXT914 quinazolinone calcilytics within the CaSR transmembrane domain using Glide Extra Precision (XP) mode. GlideScores are indicated for each pose. Residues involved in calcilytic binding are shown in blue. Calcilytic molecules are shown in orange with oxygen and nitrogen heteroatoms shown in red and blue, respectively. H-bond interaction between Trp818 and the calcilytic molecules are represented by dashed lines.
