# Supplementary material for: Characterization of quinazolinone calcilytic therapy for autosomal dominant hypocalcemia type 1 (ADH1)
Source: J Biol Chem. 2025 Mar 12;301(4):108404. doi: 10.1016/j.jbc.2025.108404 (PMC12001111; doi:10.1016/j.jbc.2025.108404)
Supplement: Figure S3 [file mmc3.pdf]

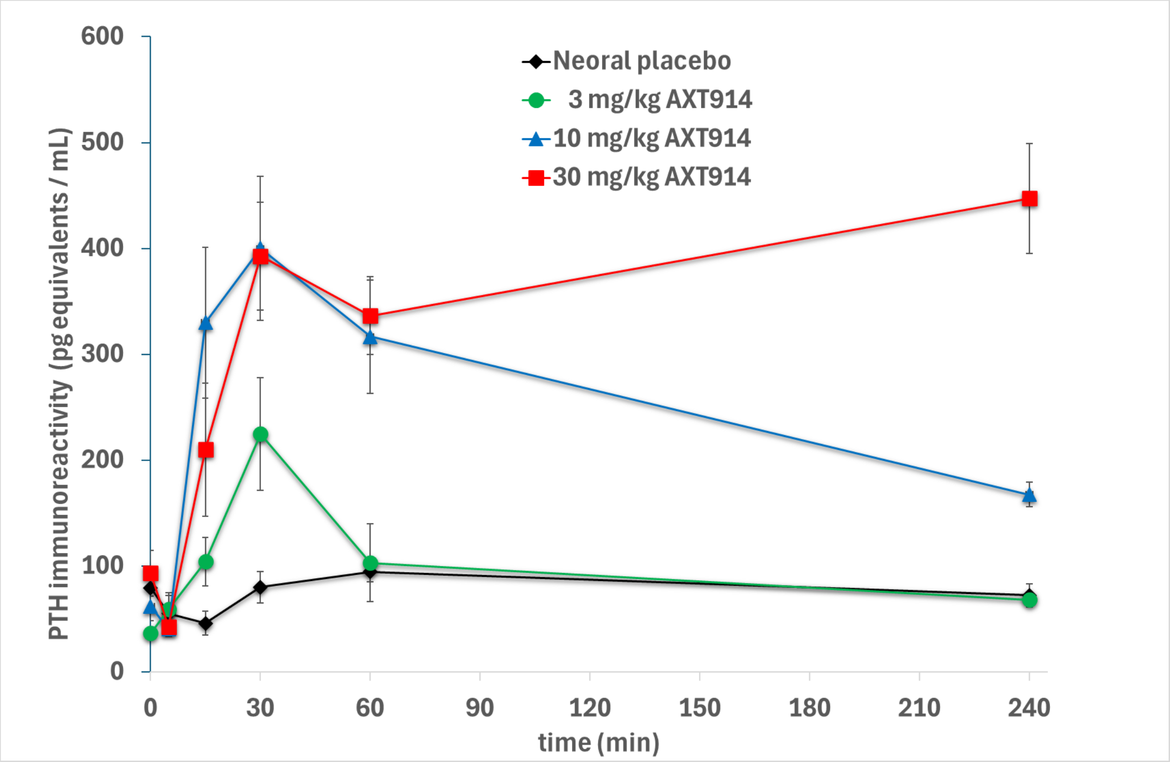

Figure S3. Effect of orally administered vehicle (Neoral placebo) or AXT914 on plasma PTH release in wild-type rats. Data is from a previously conducted study by Novartis involving n=4 adult female wild-type rats. PTH values are shown as mean ± SEM. Data provided by Novartis Pharma AG, Basel, Switzerland.
