# Supplementary material for: Characterization of quinazolinone calcilytic therapy for autosomal dominant hypocalcemia type 1 (ADH1)
Source: J Biol Chem. 2025 Mar 12;301(4):108404. doi: 10.1016/j.jbc.2025.108404 (PMC12001111; doi:10.1016/j.jbc.2025.108404)
Supplement: Table S1 [file mmc4.docx]

**Table S1. Summary of pharmacokinetic parameters for AXT914**

| Parameter | Value |
| --- | --- |
| Total clearance (mL/min/kg) | 68.7±3.2 |
| Volume of distribution at steady state (L/kg) | 12.0±0.6 |
| Terminal half-life after i.v. administration (hour) | 5.3±0.4 |
| Absolute oral bioavailability (%) | 31.1±2.5 |
| Dose-normalised C_max_ after oral administration (nM) | 54.7±9.5 |
| T_max_ (hour) | 0.3 ± 0.1 |

Pharmacokinetic properties of AXT914 were assessed in n=4 adult female wild-type rats administered either 1.0mg/kg AXT914 as an i.v. bolus or 3.0mg/kg AXT914 by oral gavage. Data provided by Novartis Pharma AG, Basel, Switzerland. Abbreviations: C_max_, maximal blood or plasma concentration; T_max_, time of maximal blood or plasma concentration.
