# Supplementary material for: Characterization of quinazolinone calcilytic therapy for autosomal dominant hypocalcemia type 1 (ADH1)
Source: J Biol Chem. 2025 Mar 12;301(4):108404. doi: 10.1016/j.jbc.2025.108404 (PMC12001111; doi:10.1016/j.jbc.2025.108404)
Supplement: Table S2 [file mmc5.docx]

**Table S2.** **Cross-reactivity of AXT914 with a panel of 52 G-protein coupled receptors, transporters and ion channels**

| Target | Cell/tissue | Binding affinity (IC_50_) |
| --- | --- | --- |
| G-protein coupled receptors |  |  |
| Adenosine 1 receptor (human) | CHO-K1 | >10μM |
| Adenosine 2a receptor (human) | HEK293 | >10μM |
| Adenosine 3 receptor (human) | CHO-K1 | >10μM |
| Adrenergic Alpha 1 receptor (rat) | Rat brain | >10μM |
| Adrenergic Alpha 2A receptor (human) | CHO-K1 | >10μM |
| Adrenergic Alpha 2B receptor (human) | CHO-K1 | >10μM |
| Adrenergic Alpha 2C receptor (human) | Sf9 | >10μM |
| Adrenergic Beta 1 receptor (human) | Sf9 | >10μM |
| Adrenergic Beta 2 receptor (human) | Sf9 | >10μM |
| Angiotensin II AT1 receptor (human) | CHO-K1 | >10μM |
| Bradykinin 1 receptor (human) | HEK293 | >10μM |
| Cholecystokinin A receptor (human) | 1321N1 | >10μM |
| Cholecystokinin B receptor (human) | HEK293 | >10μM |
| Dopamine D1 receptor (human) | L-cells | >10μM |
| Dopamine D2 receptor, long form (human) | Sf9 | >10μM |
| Dopamine D3 receptor (human) | CHO-K1 | >10μM |
| Dopamine D4.4 receptor (human) | CHO-K1 | >10μM |
| Endothelin A receptor (human) | CHO-K1 | >10μM |
| Endothelin B receptor (human) | CHO-K1 | >10μM |
| GABA-B receptor (human) | Human brain | >10μM |
| Ghrelin receptor (human) | CHO-K1 | >10μM |
| Glucagon receptor (human) | CHO-K1 | >10μM |
| Histamine H1 receptor (human) | CHO-K1 | >10μM |
| Melanocortin 3 receptor (human) | HEK293 | >10μM |
| Melanocortin 4 receptor (human) | HEK293 | >10μM |
| Muscarinic M1 receptor (human) | CHO | >10μM |
| Muscarinic M2 receptor (human) | CHO | >10μM |
| Muscarinic M3 receptor (human) | CHO | >10μM |
| Muscarinic M4 receptor (human) | CHO-K1 | >10μM |
| Muscarinic M5 receptor (human) | CHO | >10μM |
| Neuropeptide Y Y1 receptor (human) | Sf9 | >10μM |
| Neuropeptide Y Y2 receptor (human) | KAN-TS | >10μM |
| Opiate delta receptor (human) | HEK293 | >10μM |
| Opiate kappa receptor (human) | HEK293 | >10μM |
| Opiate mu receptor (human) | CHO-K1 | >10μM |
| Serotonin 5-HT 1A receptor (human) | Sf9 | >10μM |
| Serotonin 5-HT 2A receptor (human) | CHO-K1 | >10μM |
| Serotonin 5-HT 2B receptor (human) | CHO-K1 | >10μM |
| Serotonin 5-HT 2C receptor (human) | 1321N1 | >10μM |
| Serotonin 5-HT 6 receptor (human) | HEK293 | >10μM |
| Serotonin 5-HT 7 receptor (human) | Sf9 | >10μM |
| Tachykinin NK2 receptor (human) | CHO-K1 | >10μM |
| Thromboxane A2 receptor (human) | HEK293-EBNA | >10μM |
| Transporters |  |  |
| Dopamine transporter (human) | CHO-K1 | >10μM |
| Norepinephrine transporter (human) | MDCK | >10μM |
| Serotonin transporter (human) | HEK293 | >10μM |
| Ion channels |  |  |
| L-Type calcium channel, dihydropyridine (rat) | Rat brain | >10μM |
| N-Type calcium Channel (rat) | Rat brain | >10μM |
| Potassium Channel, [KATP] (rat) | Rat brain | 2.56μM |
| Nicotinic receptor (human) | IMR32 | >10μM |
| Serotonin 5-HT 3 channel (human) | HEK293 | >10μM |

Binding of AXT914 to all targets was performed as part of the Novartis *in-vitro* safety pharmacology assessment using standard radioligand binding assays. Ten or 11 different AXT914 concentrations were evaluated in each assay and generated by semi-log dilutions of an initial 10 or 30 μM concentration. Binding affinity was assessed using the half maximal inhibitory concentration (IC_50_), obtained from a normalized concentration-response 4-parameter logistic curve fitting model.

Abbreviations: 1321N1; human astrocytoma cell line; CHO, Chinese hamster ovary cell line; CHO-K1, subclone of Chinese hamster ovary cell line; HEK293, human embryonic kidney 293 cell line; HEK293-EBNA; HEK293 cells stably expressing the Epstein-Barr virus nuclear antigen (EBNA); IMR32, human neuroblastoma cells; KAN-TS, neuroblastoma cell line; L-cells, cells secreting glucagon-like peptide-1 (GLP-1); Sf9, *Spodoptera frugiperda* insect cell line. Data provided by Novartis Pharma AG, Basel, Switzerland.
